# Physical examination of the cervical spine should be conducted in female athletes with a history of concussion

**DOI:** 10.1016/j.jsampl.2026.100157

**Published:** 2026-09-18

**Authors:** Julie A. Hides, Benjamin M. Currie, Emma A. Warner, Tim J. Oostenbroek, Brett P. Dyer, Julia M. Treleaven, Felix T. Leung

**Affiliations:** 1 School of Allied Health, Sport and Social Work, Griffith University, Queensland, Australia; 2 Queensland Academy of Sport, Queensland, Australia; 3 Griffith Biostatistics Unit, Griffith Health, Griffith University, Queensland, Australia; 4 School of Health and Rehabilitation Sciences, The University of Queensland, Australia

**Keywords:** Mild traumatic brain injury, Proprioception, Neck strength, Manual examination

## Abstract

**Background:**

Female athletes may be at increased risk of injury following sports-related concussion (SRC), even after symptom resolution. Residual cervical spine impairments have been proposed as a potential contributor; however, these have not been well characterised in female athletes. This study aimed to examine whether a history of SRC was associated with clinical assessments of the cervical spine in female athletes.

**Methods:**

In this cross-sectional observational study, 156 elite and pre-elite female athletes from water polo, soccer, diving, rugby-7s and cycling/BMX were recruited. History of SRC within the previous 12 months was self-reported. Assessments included questionnaires and physical examination of the cervical spine, comprising range of motion, manual examination, joint position error (JPE) and maximal isometric neck strength. Between-group differences were examined using univariable statistical analyses.

**Results:**

Athletes with a history of SRC demonstrated a higher prevalence of reduced cervical range of motion, positive findings on manual examination, increased JPE and a history of headaches or migraines compared with athletes without concussion history. No deficits in maximal isometric neck strength were observed in athletes with a history of SRC.

**Conclusion:**

Cervical spine impairments were more commonly observed in female athletes with a history of SRC within the previous 12 months, including in athletes who did not report current neck pain. These findings suggest consideration of targeted cervical spine assessment beyond symptom-based screening in athletes with a concussion history. Whether identification and management of cervical spine impairments influence subsequent injury risk requires investigation in prospective and interventional studies.

**Key points:**

• Cervical spine impairments may be more commonly observed in athletes with a history of concussion within the previous 12-months, even in the absence of current neck pain.
• Clinical assessment of the cervical spine, including range of motion, manual examination and joint position error, may warrant consideration when evaluating athletes with a concussion history.
• Positive findings may represent residual impairments with the potential to influence awareness, movement and control of the head and neck.
• Whether identification and management of residual cervical spine impairments reduce the risk of subsequent injury requires investigation in longitudinal or interventional studies.

## 1 Introduction

Following a sports-related concussion (SRC), athletes are at an increased risk of musculoskeletal injury or another concussion [[1], [2], [3]]. While the precise reasons for this remain unknown, one possibility is that residual deficits in many subsystems affected by concussion, including cervical sensorimotor, vestibular, oculomotor and physiological systems, may be present despite symptom resolution Galea et al., 2018, [[4], [5], [6], [7]]. To understand possible reasons and potential management to prevent secondary injury post SRC, it is important to better understand the long-term impacts.

A potential area to consider is concomitant neck injury post-concussion. Previous research has identified sensorimotor changes in the cervical spine following concussion, assessed using clinical tests such as range of motion, joint position error testing, manual examination and measures of neck muscle function [5,8]. A recent cross-sectional study showed that cervical spine proprioception impairments in male and female athletes may persist for as long as three years following concussion Smulligan et al. [9]. Kinney et al. [10] reported persistent cervical sensorimotor impairments up to one year following concussion, including worse joint position error (JPE) during rotation and extension movements. These deficits were most pronounced in athletes who experienced neck pain during manual examination, who also demonstrated reduced cervical spine joint mobility. Another study reported reduced range of motion (ROM), endurance and findings on manual examination despite resolution of symptoms four weeks to six months post-concussion [5]. These findings suggest that baseline testing of cervical sensorimotor factors, such as ROM, manual examination of the cervical spine (joint mobility and pain provocation) and proprioception (JPE) may be important in athletes with a history of concussion, to detect residual deficits. Identifying residual deficits may assist clinical management, rehabilitation and return-to-sport decision-making, as well as help identify impairments that could contribute to subsequent injury risk.

Another factor that has been considered in relation to SRC is the strength of the neck muscles [11]. It has been proposed that the cervical muscles contribute to dynamic control of the head during impact, with stronger muscles helping to resist rapid head-neck motion, reduce peak head acceleration and attenuate forces transmitted to the brain [[12], [13], [14]]. Through eccentric control of head movement, the cervical musculature may also assist in absorbing and dissipating impact-related forces. Conversely, weaker neck muscles may be less capable of resisting rapid head-neck accelerations and decelerations, potentially reducing the capacity of the head-neck complex to absorb energy during head and body impacts [[15], [16], [17]]. Consistent with this proposed mechanism, prospective studies in rugby union and rugby league have reported associations between lower neck strength and increased risk of SRC [18,19], while other studies have suggested that greater neck strength may have a protective effect [20]. However, findings remain inconsistent, with some investigations reporting no association between neck strength and concussion risk [21] and others observing no differences in neck strength between athletes with and without a history of SRC Kavyani et al., 2025. Therefore, although neck strength has been widely proposed as a modifiable factor in concussion prevention, further research is required to clarify its role in both concussion risk and recovery.

Importantly, female athletes may be at increased risk of SRC compared with males due to several proposed factors, including smaller neck musculature relative to head mass, differences in head-neck anthropometrics and neuromuscular characteristics [22]. To date, residual deficits in the cervical spine have not been exclusively studied in female athletes. For these reasons, more research examining the long-term effects of SRC on the cervical spine in female athletes is required.

The aim of this study was to determine if there was an association between history of concussion and assessments of the cervical spine in female athletes from a variety of sports.

## 2 Methods

### 2.1 Design

A cross-sectional study was conducted on elite and pre-elite female athletes to investigate the association between concussion history (in the last 12 months) and assessments of the cervical spine (including ROM, manual examination, JPE and maximal neck strength).

### 2.2 Participants

Participants were aged 13–34 years and recruited from the Queensland Academy of Sport (QAS) in Queensland, Australia. Athletes listed as a national or state academy (elite) and pathway athletes (pre-elite) were eligible for inclusion. Participants were recruited across five sport programs (water polo, soccer, diving, rugby-7s and cycling/BMX) as a sample of convenience. These sports were part of the QAS program and identified as sports with a high prevalence of SRCs. All study procedures were performed in compliance with relevant laws and institutional guidelines. Written informed consent was obtained from all participants. The study was approved by the university’s human research ethics committee (HREC 2023/472).

Participants completed a questionnaire to determine demographic information, including age, height, body mass, sports participation and medical history, including history of neck injuries and concussions. For this study, a concussion history was based on a self-reported concussion (diagnosed or undiagnosed) within the last 12 months. A concussion was defined according to the 6th consensus statement for concussion in sport Patricios et al. [23].

### 2.3 Physical examination of the cervical spine

Assessments of the cervical spine were conducted by a musculoskeletal physiotherapist who was blinded to the participant’s concussion history. Screening questions were used to determine the presence of current neck pain, dizziness or headache and whether athletes had a history of neck pain, headache or migraines. The physical examination included assessment of ROM, a manual examination, and testing of cervical spine proprioception [24] and maximal neck strength [17].

Assessment of active ROM was performed in sitting, with the patient in a neutral spine position. Observations of active movements were performed in the directions of cervical spine flexion, extension, rotation and lateral flexion. The examiner applied over-pressure if the participant had full pain-free ROM. For each movement direction, the range was considered as either ‘no restriction’ (pain-free with overpressure) or ‘restricted’.

The manual examination included central and unilateral passive accessory intervertebral movements of the upper cervical (C0–C2), cervical (C3-T1) and upper thoracic (T2-T4) spinal levels. Assessment of cervical spine segmental mobility using manual examination is a commonly employed clinical approach, with acceptable reliability reported across musculoskeletal populations Lemeunier et al. [25]. Participants were positioned in prone lying while the examiner applied a posterior-to-anterior directed force. Segmental mobility (no abnormality or hypermobile/hypomobile), and pain provocation were recorded. A positive finding was considered if participants had cervical spine segmental mobility dysfunction or pain provocation greater than 2/10 on at least one vertebral level [26].

Proprioception of the cervical spine was assessed using a JPE test [17,27]. Participants were seated in front of a wall. A laser was placed on the participant’s forehead, pointing towards a standardised target with 90 cm between the target and laser. Participants were asked to sit in a neutral spine position and self-selected their neutral head-neck position. The target was placed so that the laser beam was pointing to the centre of the target. A familiarisation task was completed with eyes open, followed by testing where participants were blindfolded. The examiner aligned the laser to the centre of the target before participants completed three trials in each direction (flexion, extension, left and right rotation of the cervical spine), and participants indicated when they perceived they had returned to the centre position. The participants were repositioned with the laser at the centre of the target between attempts. Data were presented as mean absolute error in degrees for each movement direction and overall movements. A cut-off of 3.5° was also used to classify participants into normal or abnormal categories [27].

Maximal isometric neck strength was assessed using a previously published protocol [28]. A pull dynamometer (K-Pull KINVENT, France) was used to assess strength in the directions of neck flexion, extension and lateral flexion (left and right). Participants were seated on a 45 cm high bench, with their feet positioned hip width apart and flat on the floor, and arms placed by their side holding onto the bench. A warm-up was conducted prior to the testing, with participants completing self-resisted isometric contractions for 10 s at 70% maximal voluntary contraction in all four directions. A harness was then placed around their head above the ear line and across the frontal and parietal bones. Participants were instructed to maintain a neutral head-neck position and produce a maximal effort as quickly as possible. A make test was selected as it allowed gradual force generation against fixed resistance, avoiding the sudden externally imposed loads associated with break testing, which can increase post-test muscle soreness. For each direction, three repetitions of 3-s contractions were recorded with a 30-s rest between each repetition. The maximal contraction in kilogram units was recorded. Participants were excluded from maximal neck strength testing if they reported pain greater than 2/10 on the numeric pain rating scale when ROM was assessed or with manual examination as pain can influence the measurement.

### 2.4 Statistical analyses

All data were analysed using IBM SPSS Statistics software (version 30). Means and standard deviations (SD) for participant demographics (age, height, body mass, playing years) were calculated. Descriptive data for demographic variables were calculated for individual sports. To examine the association between a history of concussion in the last 12 months and cervical spine function, the following categorical and continuous variables were assessed: presence or not of positive joint signs, joint position error (in degrees) for the directions of cervical spine flexion, extension and rotation to the right and left sides and average overall, and strength of the flexor, extensor and lateral flexor muscles, flexion-to-extension (F:E) strength ratio and lateral flexion (LF) symmetry ratio (greater side/lesser side) ratios ((kilograms (kg), Newtons (N) and Newtons/kilogram (N/kg)). Univariable associations between continuous variables and concussion history in the last 12 months were examined using one-way ANOVAs for normally distributed data and the Mann–Whitney U test for non-normally distributed data. The relationship between concussion history in the last 12 months and self-reported history of symptoms, current symptoms and manual examination were assessed using cross-tabulations. Unadjusted odds ratios for the association between cervical spine variables with concussion history in the last 12 months were estimated from cross-tabulations and risk statistics. In addition, to allow clinical interpretation of results, a post-hoc analysis was conducted to determine if manual examination findings were related to the other cervical spine variables assessed in the study, using cross-tabulations. For this post hoc analysis, cervical measures were dichotomised for JPE (above or below 3.5° for overall mean absolute error) and ROM (no restriction or restriction in at least one direction of movement). Strength was not evaluated in this way due to participants with positive manual examination findings and ROM being excluded from strength testing. The significance level was set at p < 0.05 and all cases were assessed. As this was an exploratory study, no adjustments for multiple comparisons were made to avoid Type II errors [29].

## 3 Results

A total of 156 female athletes participating in water polo (n = 33), diving (n = 8), youth soccer (n = 51, professional soccer (n = 21), rugby-7s (n = 26) and cycling/BMX (n = 17) were included in the study. The athletes had a mean (SD) age of 19.7 (4.5) years, height of 169.2 (7.1) centimetres (cm) and mass of 67.7 (10.5) kilograms (kg). The average number of years playing sport was 10 (5.2) years. There were 28 (17.9%) athletes who reported a history of concussion in the last 12 months. Table 1 shows demographic data for the athletes with and without a concussion history in the last 12 months.

**Table 1:** Demographic data (age, height, mass and years in sport) for female athletes with and without a concussion history in the last 12 months.

Demographics	No concussion history (n = 128)	Concussion history (n = 28)	P Value
Age (years)	19.56 (4.4)	20.5 (5.2)	0.59
Height (cm)	169.2 (7.2)	168.9 (6.5)	0.86
Mass (kg)	68.3 (10.7)	65.3 (9.2)	0.17
Playing years	9.8 (5.0)	10.8 (6.4)	0.39

cm = centimetres, kg = kilograms.

Manual examination and JPE testing were conducted in all 156 athletes. Twenty-one athletes were excluded from strength testing (water polo = 12; soccer = 3; rugby 7’s = 3, BMX = 3). Players were excluded due to unwillingness to participate in maximal strength testing because of proximity to competition games (n = 12), current neck pain or headache (n = 8), and reduced ROM and positive manual examination findings (n = 1). Five of the 21 players (24%) who did not undergo strength testing had a history of concussion in the last 12 months. Descriptive information for the manual examination, JPE and neck strength assessments of athletes with and without a history of concussion are presented for individual sports in Appendix 1.

Between-group differences for the presence of current symptoms (neck pain, dizziness and headache), self-reported history of neck pain, headaches, and migraines, and findings for ROM and manual examination of the cervical spine for athletes with and without a history of concussion in the last 12 months are shown in Table 2. Chi-square analysis demonstrated that a history of headaches or migraines, positive findings on manual examination of the cervical spine and decreased ROM for rotation to the left (left rotation) were more prevalent in athletes with a history of concussion in the past 12 months compared with those without. In additional univariable analyses, athletes with a history of concussion in the previous 12 months had higher odds of reporting a history of headaches (OR = 3.09; 95% CI = 1.27–7.5, p = 0.013), a history of migraine (OR = 3.26; 95% CI = 1.2–8.77, p = 0.015) or having findings on manual examination of the cervical spine (OR = 2.49; 95% CI = 1.08–5.76, p = 0.03) or decreased ROM (OR = 3.76; 95% CI = 1.1–12.87).

**Table 2:** Prevalence and association of self-reported symptoms and cervical spine examination findings in athletes with and without a history of concussion.

	No concussion history (n = 128)	Concussion history (n = 28)	p-value
Current neck pain^a^			
No	119 (83.2)	24 (16.8)	
Yes	9 (69.2)	4 (30.8)	0.184ˆ
Current dizziness^a^			
No	121 (82.9)	25 (17.1)	
Yes	7 (70)	3 (30)	0.257ˆ
Current headache^a^			
No	110 (81.5)	25 (18.5)	
Yes	18 (85.7)	3 (14.3)	0.454ˆ
History neck pain^b^			
No	110 (84)	21 (16)	
Yes	18 (72)	7 (28)	0.153
History headache^b^			
No	105 (86.1)	17 (13.9)	
Yes	22 (66.7)	11 (33.3)	**0.01**
History migraine^b^			
No	114 (85.1)	20 (14.9)	
Yes	14 (63.6)	8 (36.4)	**0.015**
Findings on manual exam^b^			
No	79 (87.8)	11 (12.2)	
Yes	11 (39.3)	17 (60.7)	**0.03**
Decreased ROM rotation (L)^b^			
No	121 (84)	23 (16)	
Yes	7 (58.3)	5 (41.7)	**0.026**
Decreased ROM rotation (R)^b^			
No	117 (83.6)	23 (16.4)	
Yes	11 (68.8)	5 (31.3)	0.271
Decreased ROM LF (L)^b^			
No	111 (83.5)	22 (16.5)	
Yes	17 (73.9)	6 (26.1)	0.205
Decreased ROM LF (R)^b^			
No	110 (84.6)	20 (15.4)	
Yes	18 (72)	7 (28)	0.128

a Expected cell count less than 5.

b Chi-square test reported; ˆ Fisher’s exact test reported due to small sample size; Abbreviations: n – number/count; % percentages reported in brackets for variables for each group; ROM = range of motion; (L) = left, (R) = right. ROM of cervical flexion and extension not reported due to insufficient cell sizes for participants with decreased ROM. Odds ratios describing associations between concussion history are reported in the text and were derived from the contingency tables above.

The results for JPE and strength testing for athletes with and without a history of concussion are presented in Table 3. Results for JPE showed that there was a between-group difference for flexion of the cervical spine (p = 0.039, Cliff’s δ = −0.25). No other between-group differences related to 12-month history of concussion were observed for this measure.

**Table 3:** Neck characteristics including strength and joint position error for athletes who did and did not sustain a concussion injury during the last 12 months.

Joint position error (°)	No concussion history	Concussion history	p-value	Effect size: Cliff’s delta (95% CI)
	Median (IQR) n = 124	Median (IQR) n = 27		
- Flexion	3.13 (2.08)	4.04 (2.25)	**0.039**	**−0.25 (−0.47, −0.016)**
- Extension	2.90 (1.78)	2.88 (1.61)	0.703	−0.047 (−0.27, 0.18)
- Rotation left	3.40 (2.11)	3.09 (2.46)	0.682	−0.051 (−0.30, 0.21)
- Rotation right	3.54 (2.08)	3.50 (1.41)	0.602	0.065 (−0.17, 0.29)
- Absolute error	3.51 (1.27)	3.53 (1.70)	0.363	−0.11 (−0.35, 0.13)

**Neck strength**	**Mean (SD) n = 112**	**Mean (SD) n = 23**	**p-value**	**Effect size: Cohen’s *d* (95% CI)**

**Strength (kg)**				
- Flexion	13.50 (3.60)	13.44 (4.26)	0.942	0.16 (−0.28, 0.60)
- Extension	20.72 (3.57)	21.92 (6.32)	0.208	0.02 (−0.42, 0.46)
- LF left	15.30 (3.33)	15.94 (4.73)	0.435	0.23 (−0.21, 0.67)
- LF right	14.85 (3.50)	16.22 (4.89)	0.115	0.16 (−0.28, 0.60)
**Strength (N/kg)**				
- Flexion	1.99 (0.56)	2.07 (0.59)	0.558	0.14 (−0.30, 0.58)
- Extension	3.06 (0.56)	3.35 (0.77)	**0.033**	**0.43** (0.01, 0.85)
- LF left	2.26 (0.51)	2.48 (0.75)	0.092	0.34 (−0.10, 0.78)
- LF right	2.19 (0.53)	2.50 (0.69)	**0.017**	**0.50** (0.06, 0.94)
**Strength ratio**				
- F/E	0.65 (0.14)	0.61 (0.12)	0.216	0.32 (−0.12, 0.76)
- LF	1.12 (0.14)	1.17 (0.15)	0.113	0.35 (−0.09, 0.79)

**°** = degrees; IQR = interquartile range; absolute error = mean of the four directions; SD = standard deviation; kg = kilograms; N/kg = Newtons/kilogram; LF = Lateral Flexion; F/E = flexion/extension; LF = stronger side/weaker side.

Results for strength testing of the neck muscles showed no evidence of lower maximal isometric neck strength in athletes with a 12-month history of concussion. Contrary to our hypothesis, athletes who had a history of concussion had greater neck extension (N/Kg; p = 0.033, Cohen’s *d* = 0.43) and lateral flexion (right) strength (N/kg; p = 0.017, Cohen’s *d* = 0.5). However, these did not exceed previously published MDC values (extension: 8.95 kg; 1.1 N/kg) and lateral flexion right: 8.96 kg; 1.1 N/kg [28]. There were no significant between-group differences for the other strength variables assessed.

Results of the post-hoc Chi-square test demonstrated a significant association between manual examination findings and JPE above or below the 3.5° cut point averaged across all directions) (p = 0.036), and between manual examination findings and reduced ROM in at least one direction (p = 0.001).

## 4 Discussion

The findings of this study suggest that cervical spine impairments may persist in female athletes following concussion, even when symptoms have resolved, underscoring the importance of comprehensive physical examination beyond symptom-based screening. With respect to the observed relationship between a history of concussion and cervical spine related assessments, several recommendations could be made based on the results of the current investigation. Firstly, given the observed association between concussion history and a history of headaches and migraines, it would be important to ask athletes during baseline assessment if they have a history of these conditions. Obtaining a migraine history may have practical value when assessing athletes following concussion. Migraine history has been associated with increased symptom burden and prolonged recovery following concussion and may therefore help identify athletes who warrant more detailed clinical assessment. Rather than serving as descriptive information alone, a history of migraine may alert clinicians to a potential cervical contribution to headache presentation, particularly in athletes who are otherwise asymptomatic, and may prompt more detailed physical examination of the cervical spine, including assessment of joint mobility, range of motion and proprioception. While the present study did not assess the prevalence of diagnosed neck disorders, the presence of examination-based cervical impairments across multiple domains suggests that cervical spine assessment may be a useful component of the evaluation of athletes with a history of concussion, particularly those reporting headache or migraine.

Regarding the positive findings on examination of the cervical spine across several domains, the results of the current investigation suggest that cervical spine impairments may be more commonly identified in athletes with a history of concussion than in those without. This observation may be clinically relevant given that baseline cervical spine assessments are not routinely performed in many sporting environments. Notably, 86% of the athletes with a history of concussion did not report current neck pain and neither current nor previous neck pain was associated with concussion history or manual examination findings. Similar findings were reported by Kinney et al. [10] who found that although athletes reported minimal current symptoms, 60% reported pain on manual examination. Together, these findings suggest that reliance on self-reported neck pain alone may not identify all athletes with cervical spine impairments following concussion. Residual cervical spine impairments have previously been associated with post-concussion headaches and altered sensorimotor function Treleaven et al. [30]; [5]. Therefore, identifying athletes with persistent cervical spine findings following concussion may be clinically relevant and warrants further investigation in prospective studies.

Results for testing of JPE were only significant for the direction of flexion of the cervical spine, with athletes who had a history of concussion displaying greater median flexion error. Importantly, the median flexion error in the concussion group exceeded the 3.5° threshold proposed by Smulligan et al. [27], whereas the median value for athletes without a concussion history did not. In addition, 19 of 27 (70%) of athletes with a history of concussion compared with 54 of 124 (44%) of athletes without a concussion history exceeded this threshold. Differences between the present findings and those reported by Smulligan et al. [27] may reflect differences in age, sporting background and symptom status. In addition, Smulligan et al. [27] examined adolescents in the subacute phase of concussion, whereas the current investigation assessed female athletes with a concussion history up to 12 months post-injury. To our knowledge, this is the first study to report a deficit in this direction post-concussion. Whilst we are unable to explain why flexion was affected while extension and rotation were not, the findings support consideration of assessment in multiple directions when evaluating cervical proprioception, consistent with the approach used by Smulligan et al. [27]. Other studies have not assessed JPE in all directions [1,18,31]. Two of these studies found that impaired cervical rotation proprioception was associated with increased concussion risk in male contact sport athletes [1,18]. Differences in rotation JPE have also been demonstrated between recently concussed athletes and controls [27], as well as between athletes with and without a history of concussion Smulligan et al., 2024; [10]. While we assessed female athletes from several sports with differing physical demands, cervical proprioception may contribute to force attenuation through its role in positioning and controlling the head and neck during impact. This may complement the proposed role of cervical muscle strength, whereby stronger and better coordinated neck musculature may help absorb and dissipate impact-related forces by limiting excessive head acceleration. Laboratory studies have shown that optimal head, neck and torso alignment is associated with more efficient absorption of impact forces Viano and Pellman [32], providing a potential biomechanical rationale linking cervical proprioception and injury risk. Furthermore, cervical proprioception may be responsive to targeted rehabilitation interventions, suggesting potential clinical value in identifying impairments following concussion Röijezon et al. [33].

Results of strength testing in the current investigation showed that, compared with athletes without a history of concussion, athletes with a history of concussion did not demonstrate clinically meaningful deficits in maximal isometric neck strength. Although statistically significant between-group differences were observed for neck extension strength (N/kg) and right lateral flexion strength (N/kg), the magnitude of these differences did not exceed previously published MDC values [28]. Although no female-specific MDC values are currently available for this assessment, the observed between-group differences were substantially smaller than published MDC values reported in schoolboy rugby players. Therefore, the clinical significance of these findings is uncertain and they may reflect normal measurement variability. Similar findings were reported by Kavyani et al. [34], who observed no differences in neck strength between athletes with and without a history of SRC**.** Lack of a strength deficit in the 12 months following concussion may reflect recovery of strength associated with return-to-sport and training participation.

Interpretation of neck strength findings following concussion should also consider differences in assessment methodology, as testing protocols influence both the magnitude and relevance of measured strength. In the current study, maximal isometric neck strength was assessed in a seated position using a fixed dynamometer and a *make* test paradigm, reflecting voluntary isometric force-generating capacity under controlled conditions. Other studies have employed different body positions, handheld dynamometry or *break* testing, in which externally applied forces overcome the participant’s resistance, and these methodological differences may contribute to variability in findings across the literature [35]. This distinction is particularly relevant given that sport-related concussion is commonly associated with rapid, whiplash-like head–neck accelerations, particularly in sports like rugby, during which the cervical musculature functions eccentrically to decelerate head motion [36], whereas athletes from sports such as water polo, diving or cycling may experience different head-neck loading patterns. Accordingly, break testing may better reflect injury-relevant neuromuscular demands than maximal isometric strength alone [35].

While sex-related anatomical and physiological factors, such as head-neck anthropometrics, smaller neck musculature relative to head mass, neuromuscular control and hormonal influences, have been proposed to contribute to concussion risk in female athletes [22,37], other factors may also be relevant. In addition to absolute neck strength, the balance between cervical flexor and extensor strength may theoretically influence head-neck control during impact Streifer et al. [38]. Although no significant between-group differences were observed for this ratio in the current study, the interaction between muscle balance, neuromuscular control and concussion risk remains poorly understood [11]. Therefore, the current findings do not support an association between flexion-to-extension strength ratio and concussion history; however, further prospective studies are required to determine whether muscle balance contributes to injury risk independently of, or in combination with, other sex-specific anatomical and physiological characteristics.

Overall, the findings of this exploratory study suggest that cervical spine impairments may be more commonly observed in female athletes with a history of concussion than in those without a recent concussion history. These findings were observed across several domains of cervical spine assessment, including range of motion, manual examination findings and cervical proprioception. However, given the modest sample size, cross-sectional design and exploratory univariable analyses, the observed associations should be interpreted with caution. While cervical spine impairments may contribute to altered awareness, movement and control of the head and neck, the present study cannot determine whether these findings are a consequence of concussion or whether they contribute to future injury risk. Larger prospective studies are required to confirm or refute these findings and to determine whether identification and targeted management of cervical spine impairments influence subsequent injury incidence or clinical outcomes.

Limitations of the current investigation include reliance on self-reported concussion history, which may be subject to recall bias. Further, this study utilised routinely collected data, which enabled an exploratory analysis to provide a preliminary investigation of associations; however, the modest sample size may have resulted in lower precision and an increased type 2 error rate. The univariable analytical approach did not allow adjustment for potential confounders such as age, sport-specific exposure, migraine history, or current symptoms, and therefore the observed associations should be interpreted as exploratory. In addition, the cross-sectional design precludes inferring causality. Although the analysis focused on concussions occurring within the previous 12 months, it is possible that some athletes classified as having no concussion had sustained earlier concussions, and residual cervical spine impairments beyond this timeframe cannot be excluded. Future adequately powered longitudinal studies using causal inference methods examining cumulative concussion exposure and time since injury are required to further clarify these relationships. Athletes may also choose not to disclose previous SRCs for various reasons. Other assessments of cervical spine function, such as cervical muscle endurance, neuromotor control, dynamic head-neck control and cervical movement quality, were not conducted and may have identified additional impairments. Furthermore, there may have been confounding variables in the previous 12-month period, such as athletes receiving medical or other treatments in addition to that provided by the medical staff. While athletes were recruited from five sports, the number within each sport was not sufficient to compare the effect of participation in different sports. Finally, the exploratory univariable analyses did not account for clustering by sport or allow adjustment for potential confounders such as age, sport-specific exposure, migraine history, or current symptoms, due to concerns about model stability. Therefore, the observed associations should be interpreted as exploratory.

## 5 Conclusion

Currently, there are limited studies that have included cervical spine screening examination by trained healthcare professionals when investigating athletes with a history of concussion. In this study, female athletes with a history of concussion were more likely to demonstrate positive findings on selected cervical spine assessments, including range of motion, manual examination and proprioceptive testing, than athletes without a recent concussion history. These findings suggest that cervical spine assessment may be a useful component of a comprehensive post-concussion evaluation, particularly in athletes with a history of headache or migraine. Cervical spine impairments may remain unidentified if a patient interview alone is used when screening athletes with a history of concussion. Larger prospective studies are required to determine whether these findings represent persistent concussion-related impairments and whether their identification and management influence clinical outcomes or subsequent injury risk.

## Confirmation of ethical compliance

All study procedures were performed in compliance with relevant laws and institutional guidelines. Written informed consent was obtained from all participants. The study was approved by the university’s human research ethics committee (HREC 2023/472).

## Ethics declaration

Written informed consent to take part in the study and to publish the article has been obtained from all participants or their legal representatives. The privacy rights of participants have been observed.

This study was performed in compliance with relevant laws, regulatory frameworks and guidelines where the research took place. This study was approved by the Griffith University HREC. (Approval No. HREC 2023/472)

## Funding declaration

This study was funded by a Sport Performance Innovation and Knowledge Excellence Research Grant from the Queensland Ambulance Service.

## Declaration of competing interest

None.

## Figures and Tables

**Table:** 

	Total	No concussion history						Concussion history					
		Water polo	Diving	Football (Youth)	Football (Adult)	Rugby 7s	Cycling & BMX	Water polo	Diving	Football (Youth)	Football (Adult)	Rugby 7s	Cycling & BMX
Number of athletes n (%)	156 (100%)	27 (82%)	7 (88%)	40 (78%)	20 (95%)	19 (73%)	15 (88%)	6 (18%)	1 (13%)	11 (22%)	1 (5%)	7 (30%)	2 (12%)
**Cervical spine manual examination**													
Positive finding n (%)	66 (42%)	15 (56%)	3 (43%)	11 (28%)	6 (30%)	8 (42%)	6 (40%)	5 (83%)	1 (100%)	6 (55%)	1 (100%)	5 (72%)	1 (50%)
**Cervical joint position error (degrees)** Mean (SD)													
Right	3.89 (1.61)	4.39 (1.56)	4.10 (2.53)	3.86 (1.40)	3.80 (1.60)	4.08 (2.15)	3.23 (1.09)	3.68 (1.23)	3.43 (0)	4.05 (1.14)	5.02 (0)	3.48 (2.23)	2.25 (1.05)
Left	3.78 (1.78)	4.14 (1.60)	3.19 (1.50)	3.95 (1.77)	3.51 (1.86)	3.70 (1.61)	2.80 (0.82)	4.76 (2.09)	2.44 (0)	4.25 (2.65)	1.54 (0)	4.50 (2.23)	2.59 (0.42)
Extension	3.41 (1.62)	3.34 (1.91)	3.30 (1.20)	4.00 (1.57)	3.14 (1.27)	3.12 (1.45)	2.48 (1.17)	3.00 (1.03)	2.65 (0)	4.37 (2.53)	4.84 (0)	3.10 (0.84)	2.35 (0.17)
Flexion	3.72 (1.79)	4.14 (1.85)	3.05 (1.88)	3.81 (1.70)	3.61 (2.22)	3.27 (1.26)	2.70 (1.49)	4.31 (2.55)	2.74 (0)	4.88 (1.79)	2.36 (0)	4.00 (1.23)	3.46 (0.82)
**Maximal isometric neck strength (N/kg)** Mean (SD)													
Flexion	2.00 (0.57)	1.78 (0.43)	2.92 (0.48)	1.94 (0.45)	2.13 (0.64)	1.85 (0.62)	1.96 (0.43)	1.50 (0.74)	2.30 (0)	2.22 (0.53)	2.03 (0)	1.90 (0.65)	2.59 (0)
Extension	3.11 (0.61)	2.75 (0.47)	3.95 (0.50)	3.12 (0.54)	2.97 (0.47)	3.03 (0.61)	3.03 (0.33)	2.54 (0.91)	3.81 (0)	3.50 (0.70)	3.10 (0)	3.42 (0.92)	3.62 (0.30)
Lateral flexion (right)	2.25 (0.57)	1.99 (0.40)	2.95 (0.51)	2.17 (0.51)	2.12 (0.46)	2.30 (0.62)	2.08 (0.41)	1.62 (0.66)	3.51 (0)	2.62 (0.45)	2.08 (0)	2.51 (0.82)	2.93 (0.48)
Lateral flexion (Left)	2.30 (0.56)	2.09 (0.43)	3.06 (0.64)	2.26 (0.43)	2.20 (0.50)	2.36 (0.52)	2.06 (0.42)	1.65 (0.74)	2.85 (0)	2.68 (0.57)	1.90 (0)	2.50 (0.10)	2.83 (0.15)

SD = standard deviation; N/kg = Newtons/kilogram.
